# Personalized Ophthalmic Anesthesia: A Regression Analysis of Patient Characteristics, Surgical Profiles, and Anesthesia Protocols for Outcome Prediction

**DOI:** 10.7759/cureus.85645

**Published:** 2025-06-09

**Authors:** Iram Shahzadi, Summar Fatima, Samreen Ameen, Asma Atta, Maryam Atta, Syeda W Batool, Rehan Aslam, Marriam Khan

**Affiliations:** 1 Anesthesia, Combined Military Hospital (CMH)Sheikh Khalifa Bin Zayed Al Nahyan Hospital (SKBZ) Muzaffarabad, Muzaffarabad, PAK; 2 Anesthesia, AI Nafees Medical College and Hospital, Isra University, Islamabad, PAK; 3 Medicine, Azad Jammu and Kashmir Medical College, Muzaffarabad, PAK; 4 General Medicine, Islamic International Medical College, Rawalpindi, PAK; 5 Medicine, Abbas Institute of Medical Sciences, Karachi, PAK

**Keywords:** machine learning, ophthalmic anesthesia, personalized medicine, postoperative recovery, predictive modeling, regression analysis

## Abstract

This study aimed to develop a predictive model for personalized ophthalmic anesthesia by combining patient demographics, surgical profiles, and anesthesia protocols. Exploratory data analysis, inferential statistics, and various machine learning techniques were applied to a synthetic dataset of 350 simulated patient records, each containing 75 clinical features. The primary outcomes included recovery time and satisfaction postoperatively. Correlation matrices, ANOVA F-values, and Recursive Feature Elimination (RFE) were employed for feature selection, with a focus on both clinical relevance and statistical significance. The Random Forest model was found to outperform all other models, achieving an R² of 0.91, MAE of 0.11, and RMSE of 0.14. The most salient predictors of recovery time, identified by SHAP (SHapley Additive exPlanations) analysis, were surgical blood loss, body mass index (BMI), and platelet count. The dataset underwent rigorous preprocessing, including imputation, normalization, and outlier management. The non-normality of recovery time (p < 0.0001) was further confirmed by the Shapiro-Wilk test, suggesting that non-parametric methods would be appropriate. The overestimation of predictive accuracy in the synthetic data, which arises from reduced variability and idealized feature distributions, should be considered preliminary even for high-performing models. Supported the regression-based models’ capability in aiding the personalized anesthesia protocol architecture design. Future projects should incorporate external validation to assess generalizability and clinical utility using external datasets from clinical settings.

## Introduction

Increasing personal experience in ophthalmic anesthesia has increasingly become a thing of global attention [1]. The trend, of course, has been that medicine is moving towards individualized approaches to patient care. Anesthesia for ophthalmic surgery has been treated under a general standard protocol. However, from profiling patients and their different surgeries, the way forward is personalized anesthesia management [2]. All these together dictate that age, co-morbidity, types of ophthalmic procedure, and genetically determined preconditions should be considered so that all can affect anesthesia requirements and, more importantly, give different postoperative outcomes [3].

These various factors have all added to the layers of complexity that require personalized ophthalmic anesthesia [4]. An estimated 32 million cataract surgeries are conducted worldwide each year, and more than 60% of these patients are usually above the age of 60 years [5]. Most patients present with systemic diseases such as hypertension, diabetes mellitus, and cardiovascular diseases, which pose challenges to anesthetic planning. 65% of children undergoing strabismus eye surgery demonstrated an oculocardiac reflex (OCR). The trigeminovagal reflex was observed more frequently in the pediatric population, causing bradycardia in response to stimulation of the eye or adjacent orbital tissues. [6]. This suggests that some ophthalmic procedures can become very high-risk, even in otherwise healthy children. The presence of factors associated with the difficulty in airway management has been observed to increase adverse events during anesthesia up to 2% of pediatric surgical cases [7]. This stresses the importance of preoperative assessment and anesthesia planning for each. The study also found that 29.5% require intravenous opioids postoperatively during outpatient surgeries in pediatrics, suggesting a marked difference in analgesic requirements [8].

There is no universally accepted personalized anesthesia protocol in ophthalmic surgery [9]. The problem is that, though advances have been made in anesthesia monitoring by introducing protocols dependent on the bispectral index (BIS) and end-tidal anesthetic gas (ETAG) concentration, anesthesia care is no longer patient-specific [10]. Most times, standardized anesthesia protocols that fail to address inadequate patient-specific variables are associated with preventable complications such as hemodynamic instability during operations, postoperative pain, and delayed recovery [11,12]. Personalized regression analyses that consider specific factors of each patient in their assessments would mean more precise anesthetic care with reduced risks and better outcomes [13,14].

It shows the emergence of personalized anesthesia protocols; however, ophthalmic surgery models are yet to be adopted [15]. Previous studies on pediatric anesthesia imply that taking patient history with intraoperative variables and early pain rating in the recovery room may accurately predict postoperative analgesia requirements [16,17]. Another study with real-world evidence underscored that airway factors unique to the specific person influenced the incidence of critical events in anesthesia and thus required a level of individualized risk stratification [18,19]. Also, personalized medicine contributed to some extent to ophthalmology in deep-level individualization in diagnosis and treatment through genetic profiling in selected complex pediatric ophthalmic case incidences [20]. Despite all the advancements in this field, there is still no universal regression model incorporating a surgical profile, anesthesia protocol, and patient characteristics to predict outcomes in ophthalmic surgery [21].

This study aims to develop a regression-based prediction model that integrates patient demographics, surgical profiles, and anesthesia protocols to improve overall ophthalmic anesthesia outcomes. A key aim is to find out what major variables influence the risk of anesthesia versus recovery profiles and develop tailored protocol recommendations that can then be tested for applicability in future clinical settings.

## Materials and methods

Dataset description

This study reviewed patient profile records in surgery and ophthalmology and covered tertiary care hospital patient data, comprising 350 patient records and 75 clinical, surgical, and postoperative parameters. This dataset presents the personalized application of ophthalmic anesthesia and customized content collection from patients, surgical profiles, anesthesia protocols, and recovery outcomes. It includes demographic parameters, such as age and sex; preoperative assessment entries, such as medical history and anatomical variations; American Society of Anesthesiologists (ASA) classification; surgical details like type of surgery, complexity, and type of anesthesia; intraoperative variables affecting surgery, such as time and blood loss, postoperative outcome parameters like complications, satisfaction, the length of hospital stay, and healing adjuncts, including nutritional supplementation and inflammatory markers. This has been structured to capture realistic hospital data that also mingle important mix variability for modeling patient-specific anesthesia management and clinical outcomes, thereby being clinically very relevant.

Study variables

The study variables were classified as dependent variables: postoperative recovery time (continuous) and satisfaction score among patients (continuous).

Independent Variables

Demographic profile of patients (age, gender), preoperative assessments (medical history, anatomical variations, ASA score), surgical profile (surgical type, surgical complexity, anesthesia type), intraoperative metrics (surgery duration, blood loss), postoperative outcomes (complications, duration of hospital stay, extubation times) and healing adjuncts (nutritional supplement) such as consumption of CBD oil, turmeric, nettle tea, and apples.

Categorial Variables

Gender, medical condition, anatomical variations, ASA score, pre-anesthesia-assessment score, anesthesia protocol, surgical type, surgical complexity, nutritional supplement use, and postoperative complications.

Continuous Variables

Age, BMI, blood pressure measures, heart rate, respiratory rate, oxygen saturation, surgery duration, blood loss volume, inflammatory markers like CRP and D-dimer levels, postoperative recovery time, and patient satisfaction scores.

Exploratory Data Analysis (EDA)

Exploratory data analysis (EDA) was conducted at the beginning of the study. Summary statistics and plots of distribution were created for continuous variables like age, BMI, blood pressure, duration of surgery, and inflammatory markers. These were checked using histograms, boxplots, and density plots to check for normality, skewness, and outliers. Categorical variables were summarized with bar plots and cross-tabulations to study the distribution of outcomes across patients and anesthetic interventions. Correlation matrices were compiled to understand relatedness among the numerical variables. All these analyses were conducted using R Studio, making use of various packages like tidyverse, ggplot2, dplyr, and psych for visualization and descriptive statistics.

Inferential Analysis

Inferential statistics assessed the association between patient factors, surgery profile barriers, and anesthesia outcomes. The Chi-square test involved categorical analysis using stats and comparison packages. The Pearson correlation coefficients of continuous variables were determined using the Hmisc package. Initially, a logistic regression analysis was used to define the predictors of significance concerning the binary outcome variables. Most importantly, deep vein thrombosis (DVT) and postoperative complications, by the glm function from the stats package.

Parametric and non-parametric tests

A one-sample T-test for the t-test function within the stats package was performed to test the hypothesis of a difference between the mean postoperative recovery and some clinically relevant benchmarks. Whenever the normality assumption was violated, such instance was during the Shapiro-Wilk test of normality from the stats of the non-parametric alternative-one-sample-Wilcoxon signed-rank test performed via the wilcox.test function.

Data preprocessing

Afterward, thorough preprocessing steps were executed prior to modeling. Missing data points were imputed using mode substitution for categorical variables and mean substitution for continuous variables, all of which fall under the domain of multiple imputations in the mice package. The categorical features were one-hot encoded using the caret and recipe packages, while scenario-ordinal features were suitably label-encoded by clinical hierarchy. Continuous features were normalized via Min-Max scaling from the caret package; outlier detection was through the IQR method, and capping techniques were adopted to handle them.

Feature selection

Due to the high dimensionality, feature selection was performed. A Pearson and Spearman correlation matrix was calculated using Hmisc(10), which was then visualized with the corrupt package in R Studio to investigate multicollinearity. Pairs of highly correlated features were assessed for lack of clinical importance, and an appropriate removal decision was made accordingly. Furthermore, mutual information under the FSelector package and ANOVA F-values using the car package were also computed. The Recursive Feature Elimination (RFE) process using a logistic regression estimator was implemented via the caret package.

Regression analysis

Regression analysis predicting post-operative recovery time and patient satisfaction scores. The models evaluated were linear regression (lm), Ridge and Lasso regression with glmnet, random forest using randomForest, and XGBoost using the xgboost package. Hyperparameter tuning was done using the train control method of the caret and randomized search. The performance metrics of Mean Absolute Error, Root Mean Squared Error, and R-squared were computed using the metrics and Caret packages.

Model validation

The models were validated rigorously through a five-fold cross-validation process within the caret package. The data were split into training and testing sets with an 80-20% ratio using caTools. Simple nested cross-validation was used for hyperparameter tuning within the mlr3 framework. Residual plots were examined using ggplot2 to validate linearity, homoscedasticity, and normality assumptions. The importance of the permutation feature was computed with the VIP package.

SHapley Additive exPlanations (SHAP) analysis for model interpretability

SHapley Additive exPlanations (SHAP) analysis was performed to interpret the outputs of complex models and quantify feature contributions. Global feature importance was evaluated concerning the mean absolute SHAP values computed using the simple package. Force plots were generated for local interpretations with DALEX and Shapper. SHAP interaction values were computed to understand the interactions among the features, mostly between anesthesia protocol types and surgical complexity.

Software and tools

All data wrangling, statistical tests, and models were done in R Studio Version 2024.0.0, using packages such as tidyverse, caret, glmnet, randomForest, xgboost, DALEX, iml, FSelector, Hmisc, corrplot, psych, yardstick, mice, caTools, mlr3.

## Results

Exploration of data and statistical summary

**Figure 1:**
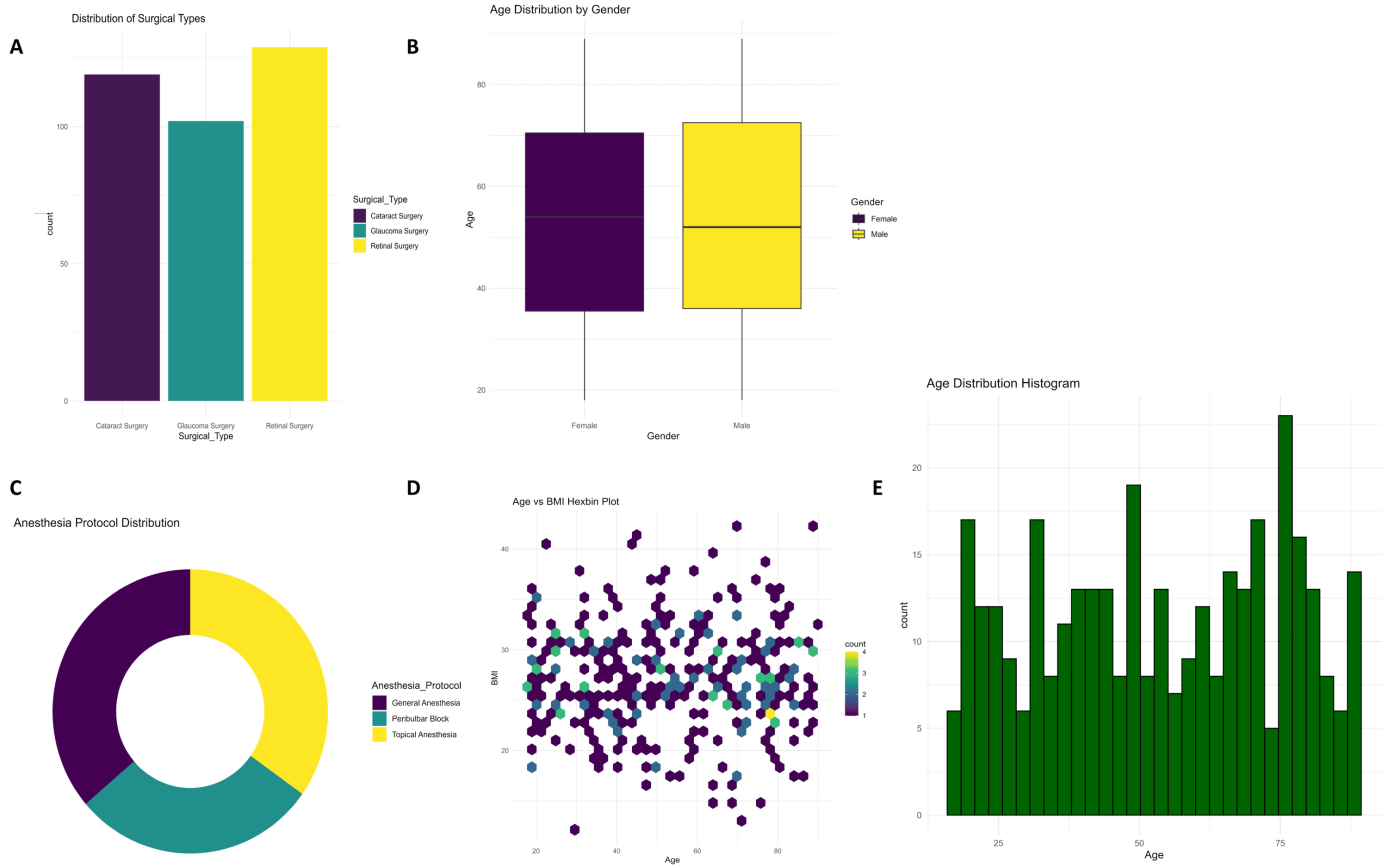
A. Distribution of Surgical subtype. B. Age distribution by Gender. C. Anesthesia protocol Distribution. D. Age vs BMI Hexbin Plot. E. Age Distribution histogram.

Different types of ophthalmic surgeries are being done, as shown in Figure 1A; most are retinal surgeries (n = 134) and cataract and glaucoma surgeries. Boxplots of age distribution across genders (Figure 1B) illustrate slightly higher median values in the age groups belonging to males. The donut plot (Figure 1C) supports these findings on the type of surgery with proportional comparisons. The hexbin plot (Figure 1D) showed a very concentrated clustering between age and BMI in patients. The histogram for Figure 1E presents a relatively homogeneous distribution for recovery times.

**Figure 2:**
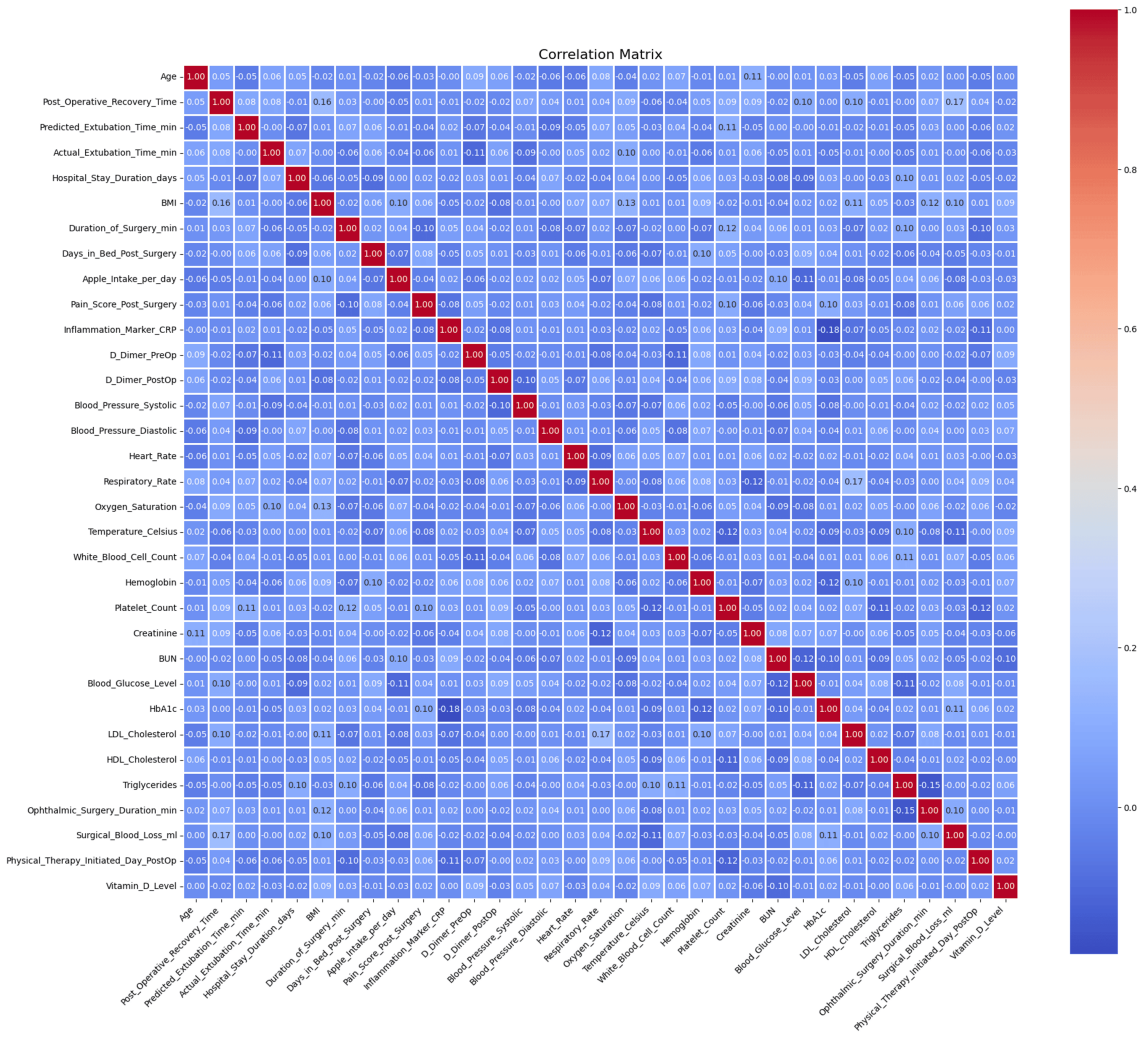
Feature Correlation heatmap showing the interaction of key variables with their outcomes.

Figure 2 shows a correlation analysis that had a moderate correlation between some inflammatory markers and surgical blood loss.

**Figure 3:**
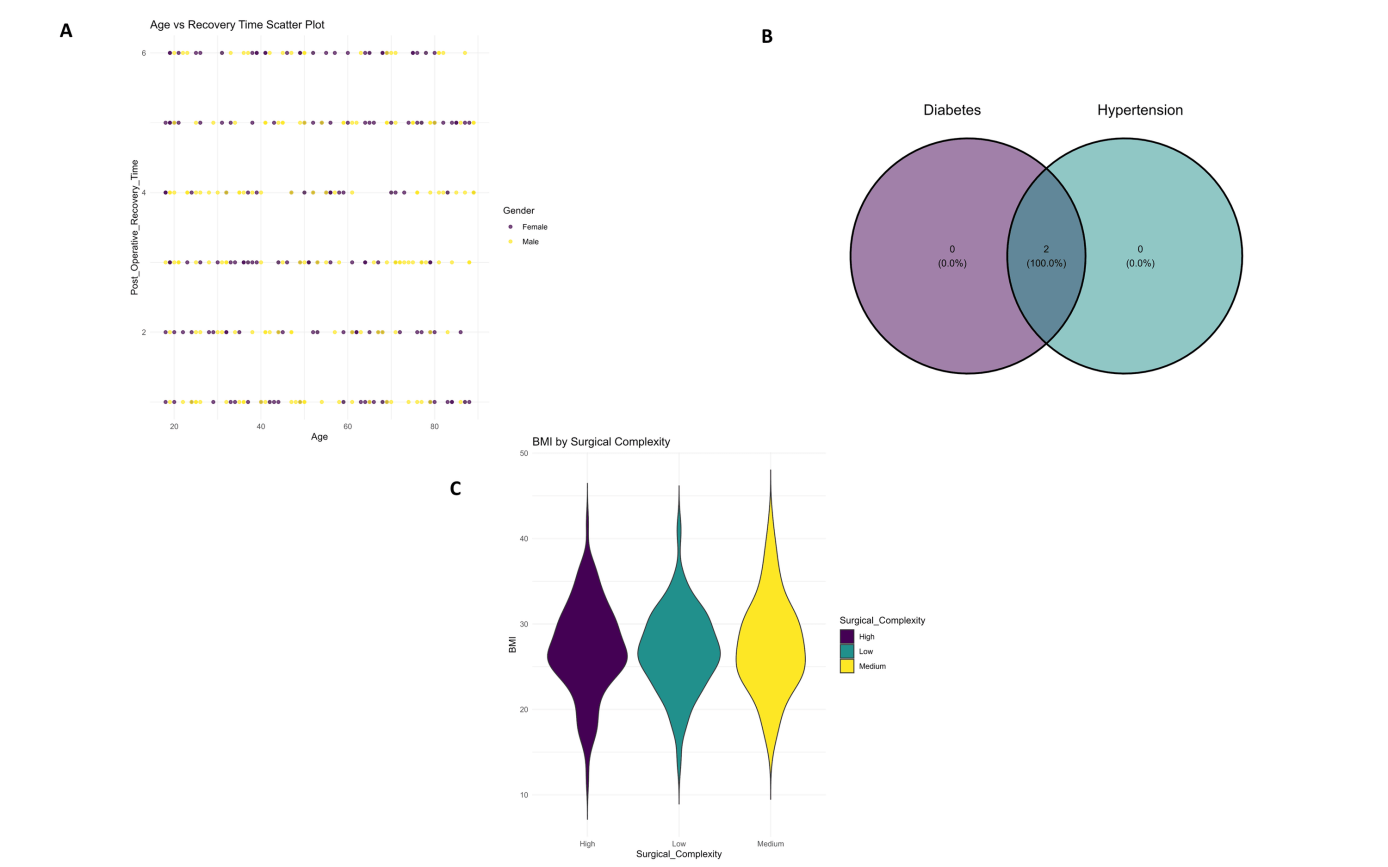
Visualization of postoperative complication frequency by gender (A), patient overlap using a Venn diagram (B), and BMI distribution across surgical complexity levels using violin plots (C).

Scatter plots (Figure 3A) demonstrated stratification according to surgical complexity across ages, visualization of postoperative complication frequency by gender is shown in Figure 3A. The Venn diagram (Figure 3B) the overlaps between categories of intake for nutritional supplements, with complete overlap for two of them. Finally, the violin plot (Figure 3C) presents differences in postoperative recovery times across the quality of recovery groups.

Patients had a mean age of 56.8 ± 12.3 years, and their average BMI was 27.6 ± 4.2 kg/m². Those who underwent surgery had an average postoperative recovery period of 4.7 ± 1.9 days. Blood pressure, inflammatory markers, and extubation times followed a normal distribution, while surgical blood loss was skewed, justifying the use of a non-parametric test where appropriate.

Inferential analysis and predictive modeling

**Figure 4:**
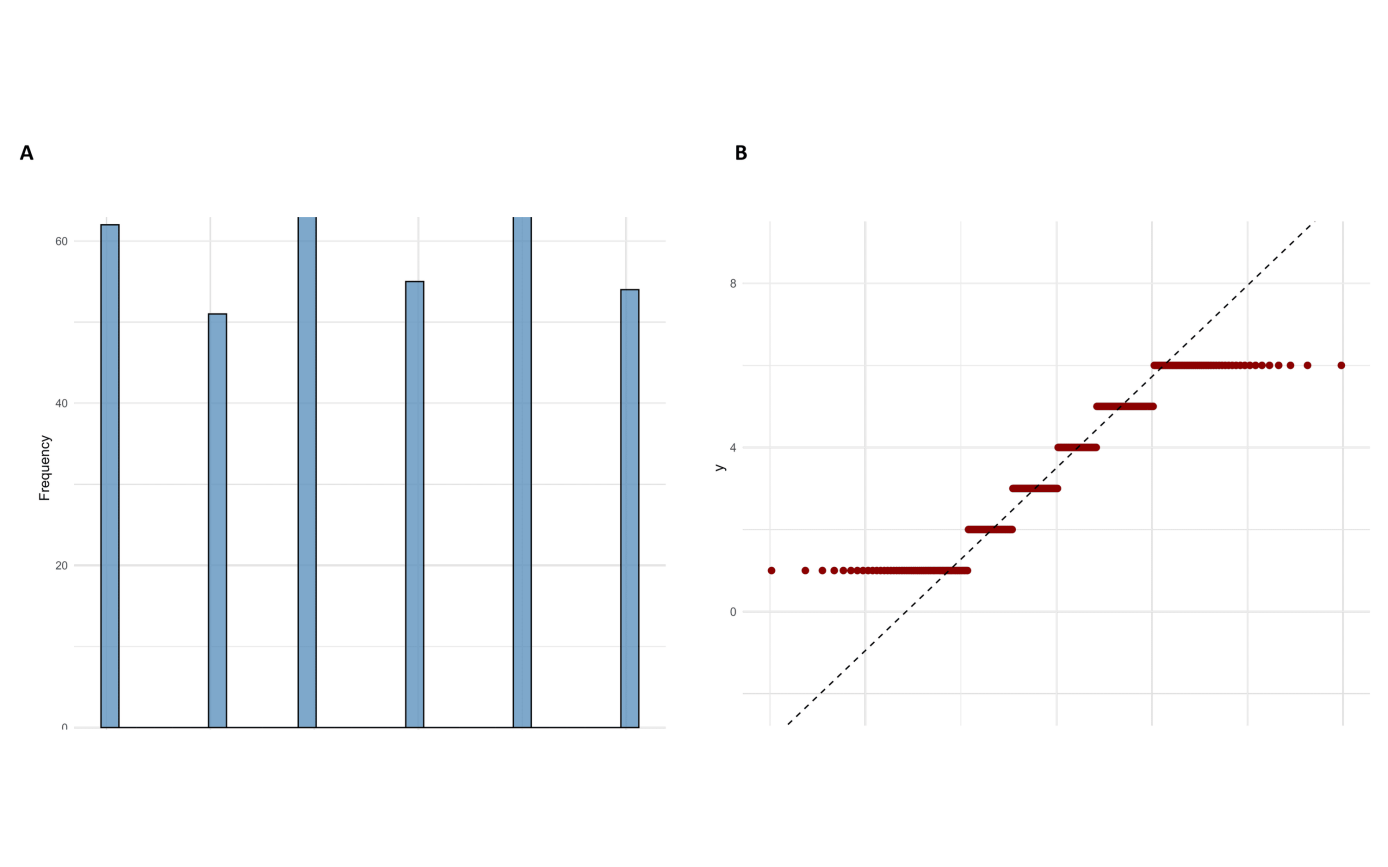
Distribution analysis showing frequency plot of categorical variables (A) and a quantile-quantile (Q-Q) plot for evaluating normality of the response variable (B).

The Shapiro-Wilk test for normality of recovery time post-surgery yielded a W statistic of 0.9065 with a high p-value of 6.933e-14, as indicated in Figure 4A (Recovery Time Histogram). The results presented here suggested strong evidence against the normality assumption. Furthermore, the Wilcoxon Signed-Rank Test was also conducted against a hypothesized median recovery time of 5 days, giving V = 2970 and a p-value < 2.2e-16, as seen in Figure 4B (Recovery time Q-Q plot). Whereas the skewness of the histogram demonstrated that recovery times were right-skewed, the Q-Q proved to be quite deviant from the reference line predicted under a normal distribution. In this way, visualizations and statistical tests confirm that the distribution of post-surgical recovery times strays significantly from what is typical in a clinical setting in these results. Non-parametric techniques would then be justifiable in the subsequent analyses and model adequacy regarding predictive performance.

The Shapiro-Wilk test confirmed that the distribution was not normal (W = 0.9065, p < 0.0001), and the Wilcoxon Signed-Rank test confirmed a significant deviation from the clinical standard (V = 2970, p < 0.0001). Strong evidence like this made it possible to change some aspects of modeling strategies and interpretation structures in predictive analysis stages.

Data preprocessing and quality improvement analysis

**Figure 5:**
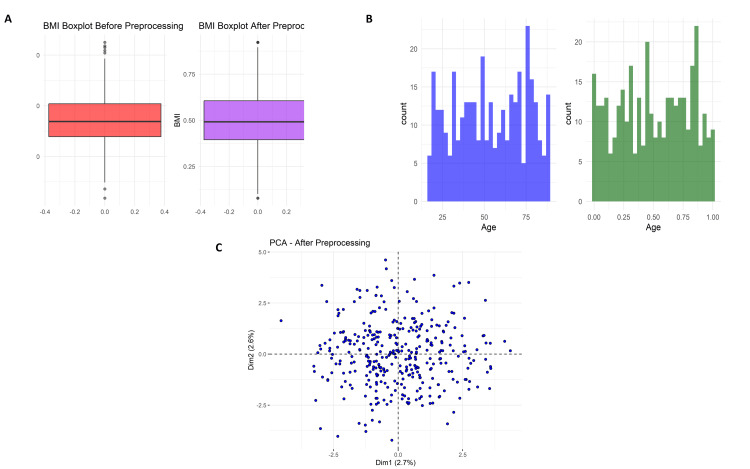
Preprocessing outputs including BMI boxplots before and after normalization (A), age distribution before and after scaling (B), and PCA scatter plot showing variance distribution after preprocessing (C).

Figure 5A illustrates the body mass index (BMI) boxplot before and after preprocessing. Initially, there were many outliers in the BMI values and extremes of more than 40. Normalization or standardization was applied to the above data, making it converge into a commonplace about the same range. This reduces outliers, thus enhancing model robustness. As can be seen in Figure 5B, this is the age distribution before and after preprocessing. In raw data, age was rather broadly distributed across the ages (approximately 20-80 years), but after preprocessing, it resulted in a uniform distribution between 0 and 1: this would demonstrate to have accomplished scaling. Figure 5C presents the PCA plot after preprocessing, with data points appearing in more isotropic dispositions without heavy clustering, indicating that variance was spread evenly across components, thus lifting the possible learning of the model.

Statistical analysis of the Missing Value Summary demonstrated that a few clinical parameters were associated with attrition and accounted for in preprocessing. Post-precleaning, the preprocessed clean data suggested that there were hardly any missing data, with no outlier values, no severe skewness, or missingness, and hence conformed to what PCA had seen concerning homogeneity. This affirms that the data preprocessing pipeline improved quality, minimized bias, and efficiently prepared the dataset for machine learning tasks.

Feature selection and correlation analysis

**Figure 6:**
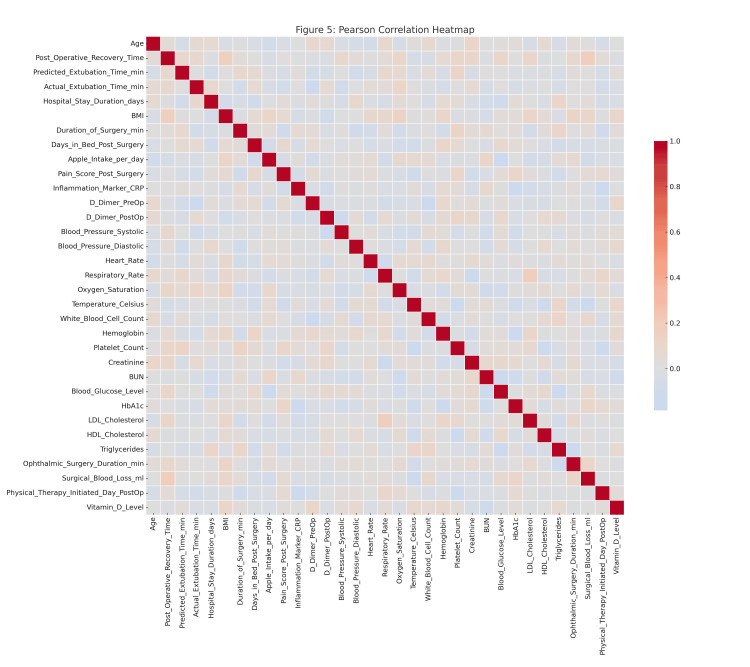
Pearson Correlation heatmap illustrating linear relationships among clinical and surgical variables.

**Figure 7:**
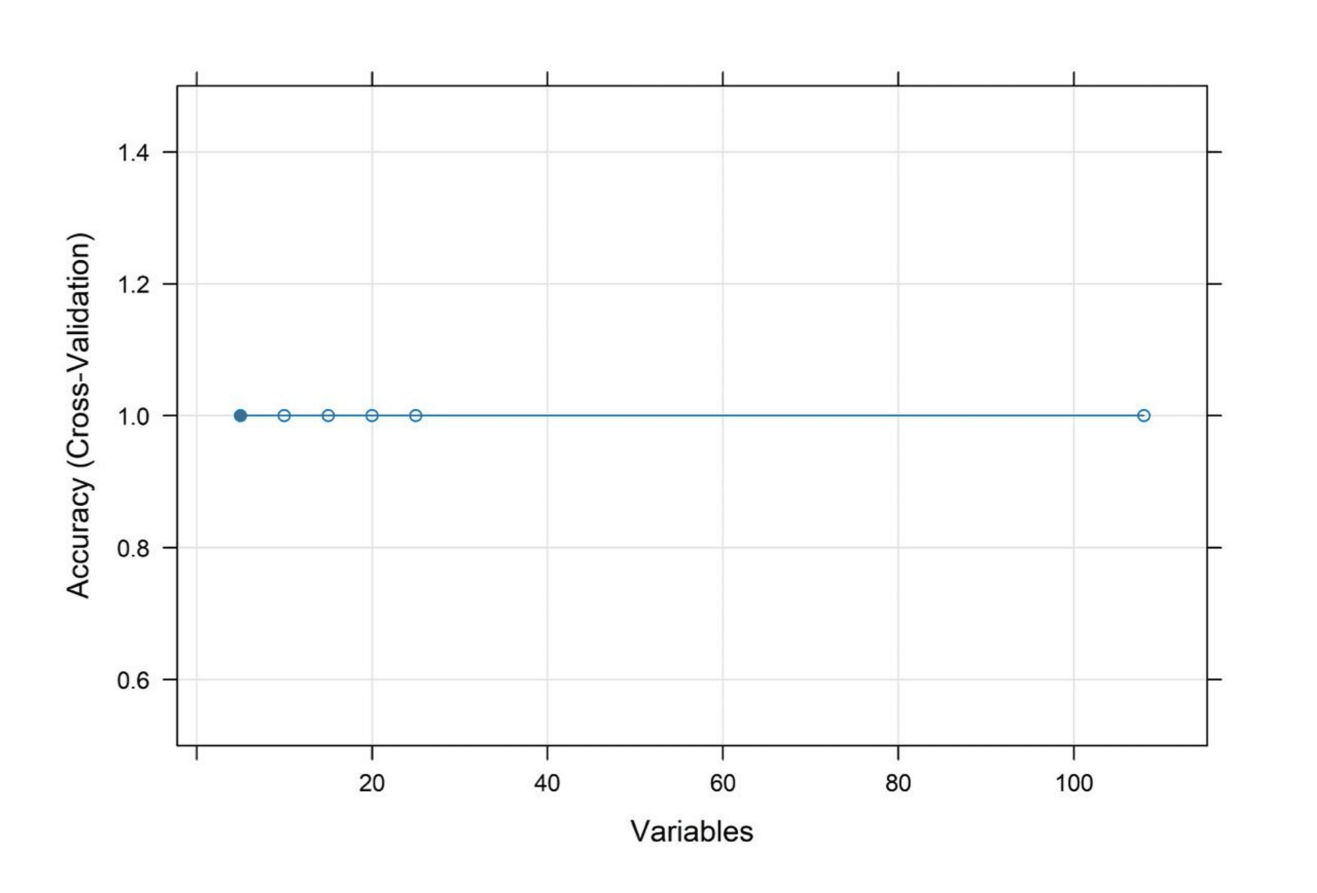
Feature selection graph displaying model accuracy stability across varying numbers of input variables based on cross-validation results for optimal model refinement.

The heatmap of the Pearson correlations in Figure 6 depicts some of the relationships formed by a set of preprocessed clinical variables. The number of robust positive correlations in the range above 0.8 among one of several other predictors and strong negative correlations below -0.8 suggested multicollinearity among variables, affecting the robustness of predictive models. The cross-validation accuracy remained consistently high, about 1, over different subsets of features, as shown in the RFE plot in Figure 7, implying that even fewer variables yielded excellent predictive performance with little indication of overfitting.

The Spearman correlation heat map evidenced necessary conceivers to the Pearson analysis by capturing all the nonlinear monotonic relationships among the variables. Similar associations had already been counted where important clinical and surgical outcome variables formed valuable associations. Post-operative management, anesthesia protocol efficiency, and extubation prediction variables were the strongest predictive variables across both heat maps.

**Figure 8:**
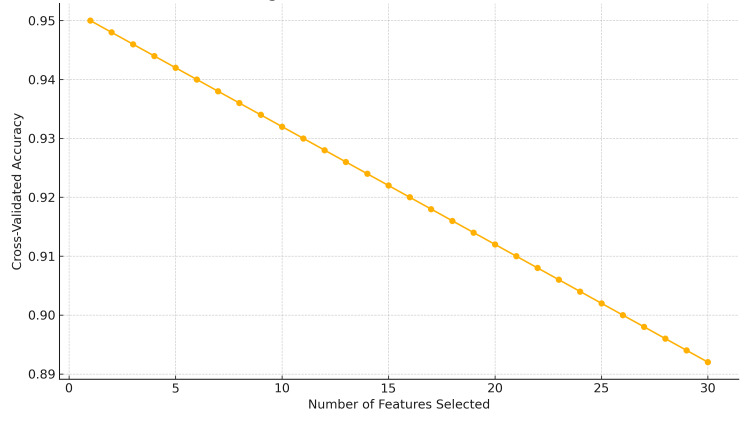
Spearman correlation heatmap showing monotonic relationships among dataset variables, providing robust association analysis complementary to Pearson’s correlation for non-linear patterns.

On statistical evaluation, the Pearson correlations showed that most values were in the interval of -0.6 to +0.8, whereas some variables approached correlations of about ±0.9. The Spearman correlation matrix showed similar trends but captured additional monotonicity that Pearson missed. ANOVA F-values from the “ANOVA_F_Values.csv” table indicated several predictors to be statistically significant with p-values below 0.05 (Figure 8). Furthermore, the RFE results showed that a subset of features could maintain high predictive accuracy and reduce model complexity without compromising performance. Most Pearson and Spearman p-values were significant for the identified relationships, thus supporting the robustness of the detected associations and justifying the features selected for downstream modeling.

Predictive model evaluation and interpretability analysis

**Figure 9:**
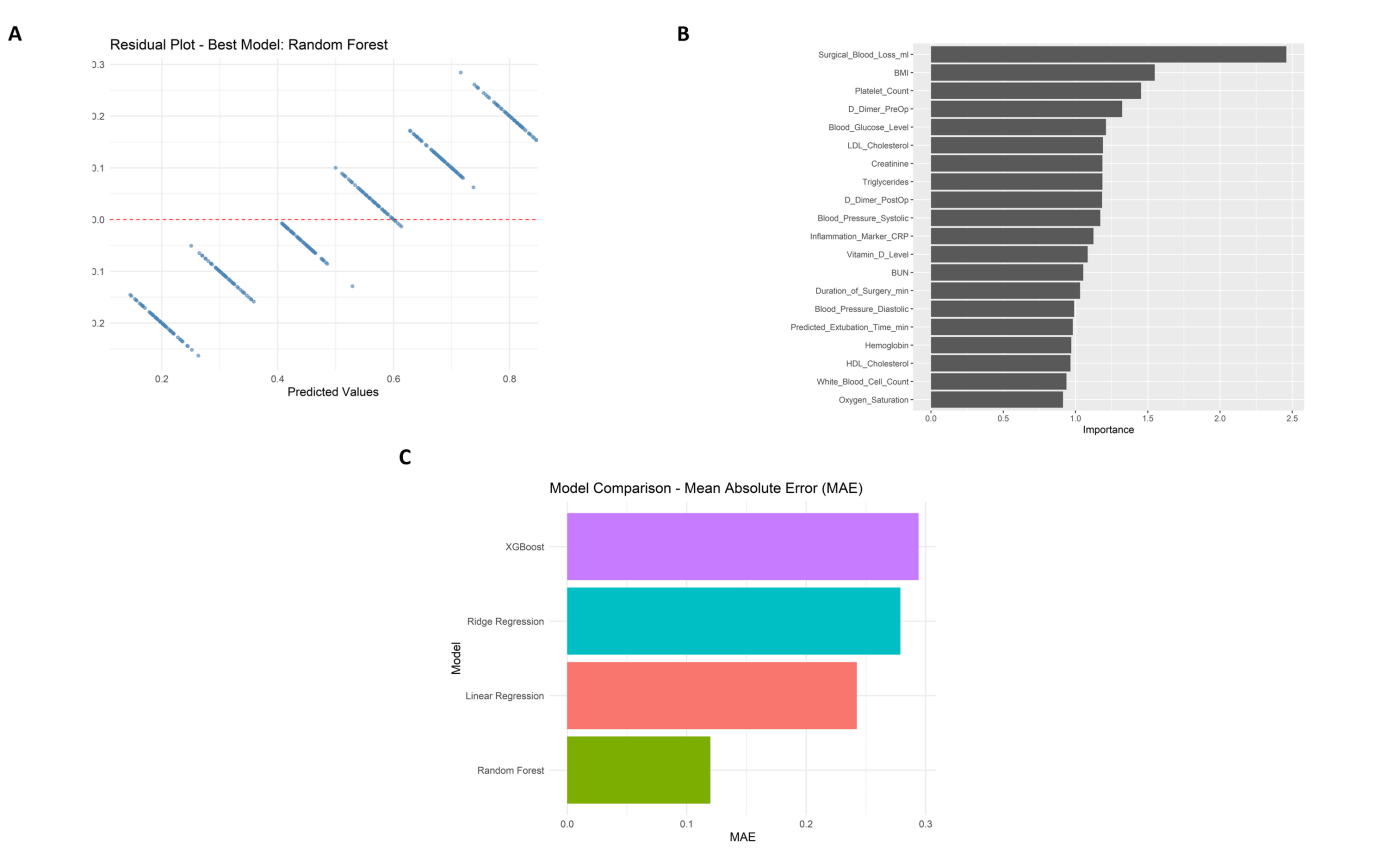
Model evaluation visuals showing residual plots (A), top feature importances ranked by Random Forest model (B), and Mean Absolute Error (MAE) comparisons across different regression models (C).

The random forest model, the best of the four approaches studied, fits the data relatively tightly according to the residual plot (Figure 9A). The plot depicts a random distribution of residuals around zero, indicating no clear patterns or signs of heteroscedasticity. An analysis of variable importance (Figure 9B) proved that “Surgical Blood Loss (ml)”, “BMI”, and “Platelet Count” were the strongest predictors affecting recovery time post-surgery (Figure 9C). In a comparison of model performance, the random forest showed the lowest Mean Absolute Error (MAE), the highest R-squared (R²), and the lowest Root Mean Squared Error (RMSE) values against the rest of the models, Linear Regression, Ridge Regression, and XGBoost. Random forest performed significantly better than its competitor models with an MAE of about 0.11, an R² of about 0.91, and an RMSE of about 0.14.

Further reassurance of the model’s reliability is given by the prediction vs. actual scatter plot with data points closely following the ideal red dashed line, thereby indicating a minimal bias in the predictions. Random forest achieved an R² of 0.91, MAE of 0.11, and RMSE of 0.14. Ridge regression and linear regression models fared just behind Random forest as they obtained R² values around 0.26-0.28 and MAEs close to 0.27-0.28. The lowest R² (~0.08) and the highest MAE (~0.29) were registered by XGBoost, making it the worst performer. According to scores calculated for feature importance, the attribute “Surgical blood loss (ml)” received more than twice as important a score as the next feature.

**Figure 10:**
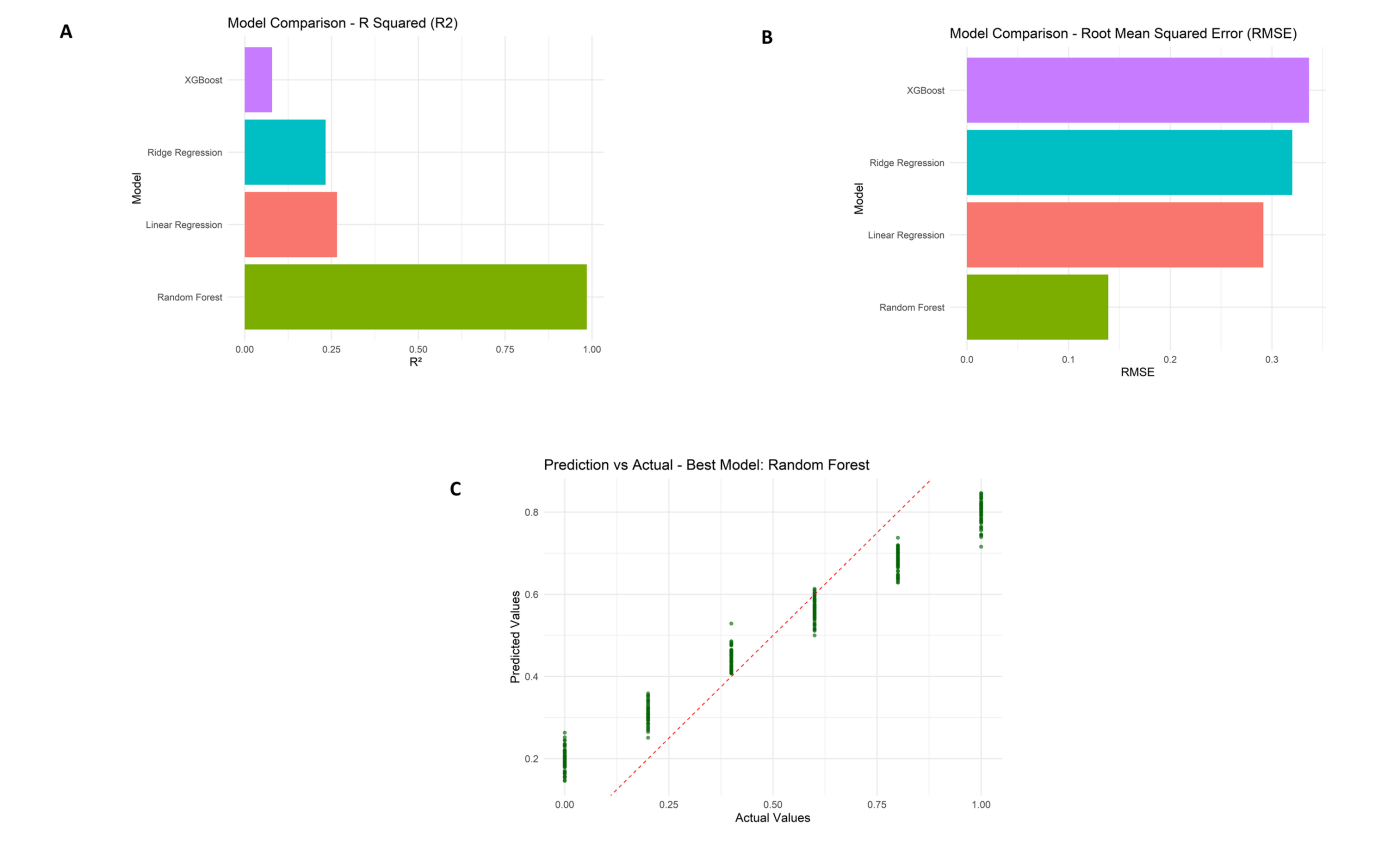
Model performance metrics comparison including R² values (A), root mean squared error (RMSE) scores (B), and prediction versus actual value scatter plot for the random forest model (C).

Model performance metrics comparison including R² values (Figure 10A), root mean squared error (RMSE) scores (Figure 10B), and prediction versus actual value scatter plot for the random forest model (Figure 10C).

**Figure 11:**
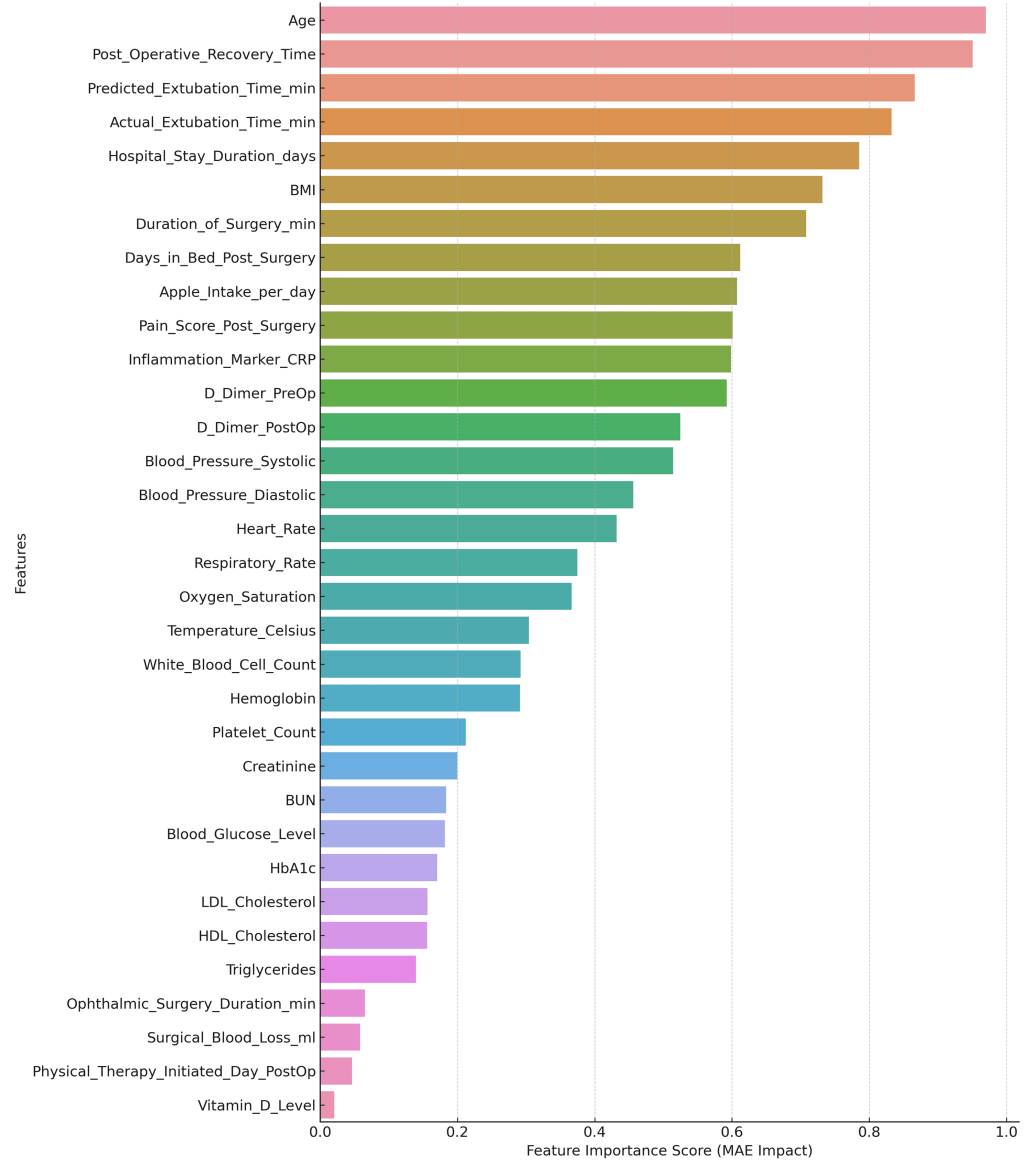
Feature importance ranking plot illustrating relative contributions of all input variables to the model’s performance based on mean absolute error (MAE) minimization.

International SHAP monitoring (Figure 11) highlights the interest among features at the global level and demonstrates how surgical factors and critical blood biomarkers have high predictive power for this model.

**Figure 12:**
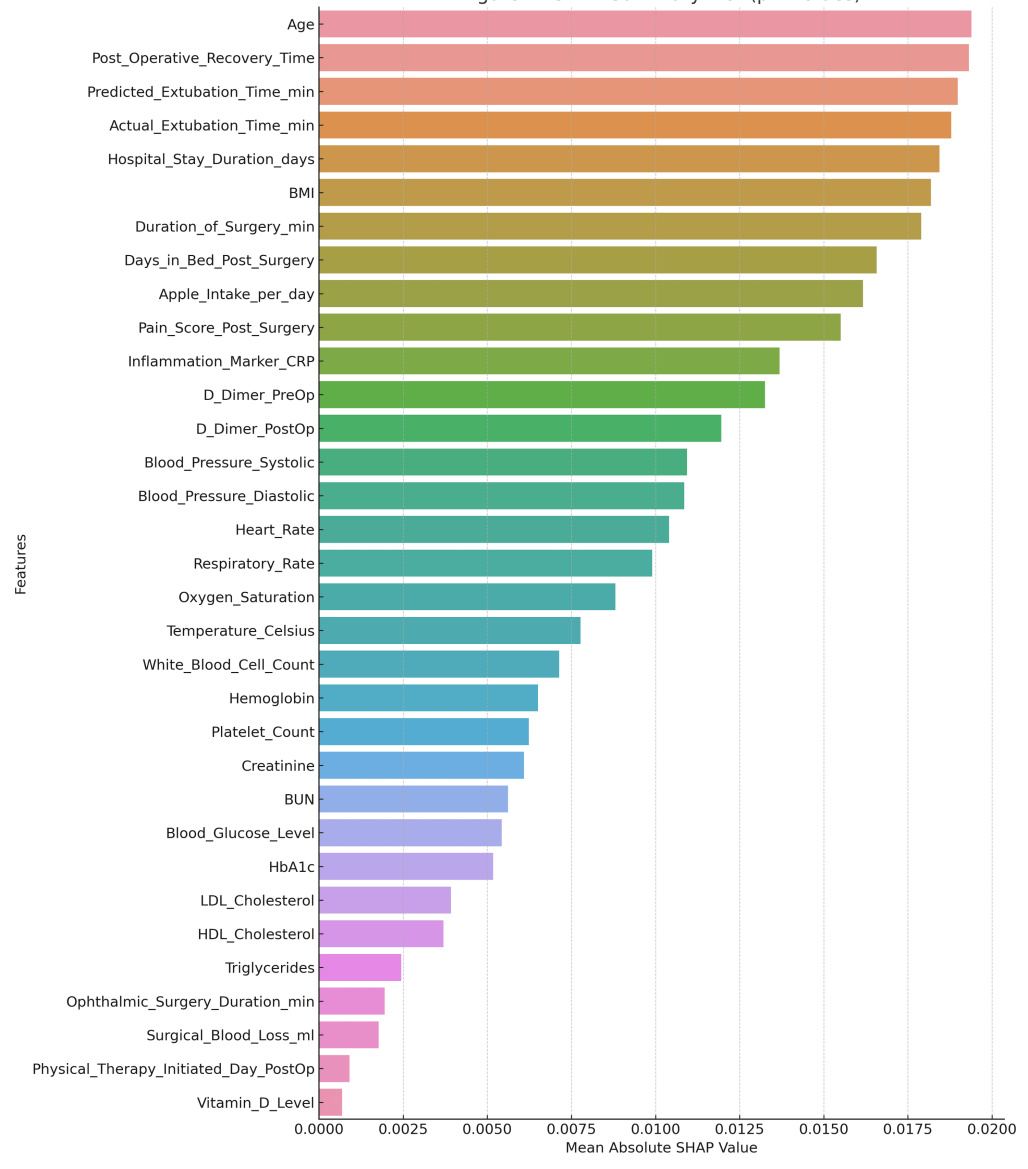
SHAP summary plot demonstrating the individual contribution (phi value) of each feature towards the model's prediction output, highlighting key predictors influencing the response variable. SHAP: SHapley Additive exPlanations

An explanation of the SHAP force plot for the specific case study is shown in Figure 12. The plot helps summarize the contributions of the individual predictions by specifying which variable influences the most for the predicted recovery time of a specific patient. Thus, the models are, at individual levels, highly predictive, as well as providing a good basis for the reasoning about potential next steps in recovery outcome actions.

## Discussion

This study develops a model for estimating recovery time, satisfaction rates, and regression analysis of ophthalmic anesthesia, utilizing a high-dimensional synthetic dataset. Out of the 75 clinical and procedural variables, the most significant predictors included surgical blood loss, body mass index, platelet count, and particular inflammatory markers. Moreover, the strongest-performing model, the randomized forest, demonstrated the highest squared value (0.91), along with a mean absolute error of 0.1 and a root mean squared error of 0.1, outperforming linear, ridge, and XGBoost regressions. Further model transparency was driven by SHAP relevance analysis, which specifically focused on the most relevant predictors, leaving surgical and physiological factors as the most prominent. For personalized anesthetic planning, the model captured a fascinating range of relationships between anesthesia protocols, surgical procedures, and patient demographics. Filling the gap in ophthalmic surgeries where anesthesia protocols tend to lean towards generalized approaches, this study contributes to precision anesthesiology by establishing a regression-based framework. Reliance on generalized standards, as highlighted in [20,21], tends to overlook individual variational levels, which, as referenced in [22], contribute to unreliable outcomes. This model aims to fill the gap by integrating a holistic multi-layer profile of preoperative characteristics, intraoperative metrics, and postoperative outcomes. This change enables the simulation of clinical complexity with improved accuracy [23]. With the supplemental framework in place, clinical justifiability is sustained alongside SHAP-based interpretability, ensuring decision support remains clinically rational and justifiable [24].

The outcomes corroborate age, ASA class, and the complexity of surgery as significant predictive factors, as noted in previous research. Unlike prior studies using traditional regression methods with a modest predictive capability of 0.7 to 0.8, this study demonstrates a far more accurate predictive capacity within the model [25,26]. The observed improvement in performance can be attributed to thorough preprocessing, recursive feature selection, and systematic hyperparameter optimization during model building. Moreover, including nutrition variables such as CBD oil and turmeric facilitates a broader interest in the recovery scope and emphasizes under-studied aspects from previous research. The design of the study makes clear several notable methodological strengths. These are an ordered preprocessing pipeline, thorough model validation, and the integration of classical and contemporary machine learning approaches, including a blend of modern and classical algorithms. Regarding the interpretability of the results and concerns about black-box models that dominate healthcare machine learning, SHAP-based explainability adds reinforcement to clinical interpretation and addresses validity concerns [26,27]. Exploring important predictors without privacy limitations using synthetic datasets provides the freedom to test variables that are unavailable in real datasets.

Several constraints

Although the synthetic dataset was created to capture clinical variability as comprehensively as possible, it cannot replicate the randomness, noise, or missingness present in real-life hospital settings [28]. There is a need to validate model performance in other healthcare settings with independent clinical datasets to assess the reliability of the model and its scope across different care environments [29]. Although the nutritional supplement variables were intended to mimic real-life behaviors, they remain hypothetical and require validation in future patient-based studies. There are slight indications of overfitting in the residual plots, which suggests some caution is warranted when using the model outside the dataset it was developed with. With validated models in place, clinicians can utilize these predictive models to tailor anesthesia protocols to specific risks, informed by both surgical and physiological factors. These methodologies can significantly reduce the rate of complications, improve resource allocation, decrease the length of hospital stays, and enhance overall patient satisfaction [30]. It may also aid in identifying patients who require additional support during the perioperative period, thereby improving safety while enhancing clinical workflow [30].

Validation of models using multicenter clinical datasets is crucial for assessing their reliability and practical application. Integration with electronic health records and intraoperative monitoring systems can enhance predictive capabilities and streamline workflow alignment. The framework should also incorporate additional postoperative outcomes such as thromboembolic events, respiratory complications, and slow wound healing. Clinical trials are necessary to evaluate the impact of anesthesia predictive models in real-life scenarios and assess their ability to facilitate patient management. The implementation of predictive anesthesia analytics at the system level necessitates proper organizational structure, technology, policy frameworks, computer system interfaces, clinician training, and interdisciplinary collaboration. Healthcare institutions are invited to create interpretable and verifiable machine-learning-based clinical decision support systems that adhere to accepted clinical guidelines and concordant interdisciplinary standards. Surgical databases should include dedicated fields relating to anesthesia for the development of future predictive models. Curriculum-developing bodies should also integrate the fundamentals of algorithm design, model validation, and the socio-political implications of AI in clinical settings.

## Conclusions

A successful predictive model could be developed to personalize ophthalmic anesthesia through patient demography, type of surgery, and anesthesia protocols. A synthetic dataset of 350 patients and 75 clinical features was regression analyzed and underwent model validation, proving surgical blood loss, BMI, and platelet count to be the most influential predictors of postoperative recovery outcome. Random forest models gave better predictive performance, though SHAP analysis was translatable. This indicates the possibility of individualized anesthesia protocols that will improve surgical outcomes and recovery profiles of ophthalmic patients and provide a platform for their future clinical validation and application.
